# 41.1 WHAT DO META-ANALYSES TELL US ABOUT CLOZAPINE’S EFFICACY AND EFFECTIVENESS FOR TREATMENT REFRACTORY SCHIZOPHRENIA?

**DOI:** 10.1093/schbul/sby014.169

**Published:** 2018-04-01

**Authors:** Dan Siskind, Steve Kisely, Rachel Land, Lara McCartney

**Affiliations:** 1 Metro South Addiction and Mental Health Service; 2 University of Queensland, MSAMHS; 3 University of Queensland; 4 University of Melbourne

## Abstract

**Background:**

Clozapine has long been considered the gold standard antipsychotic for treatment refractory schizophrenia (TRS). There have been a number of recent meta-analyses of efficacy of clozapine on psychotic symptoms and effectiveness in reducing hospitalisations that have sparked debate on the role of clozapine.

**Methods:**

Current literature regarding the efficacy of clozapine for TRS, including pair-wise and network meta-analyses of RCTs with reported outcomes of total psychotic symptoms, positive symptoms and negative symptoms were reviewed. We also examined the results of a meta-analysis of the effectiveness of clozapine on reducing hospitalisations based in RCTs and observational studies.

**Results:**

Two recent meta-analyses: Samara et al (2016), a network meta-analysis in JAMA Psychiatry; and Siskind et al (2016) a pairwise meta-analysis in BJPsych, found similar equivocal results for total psychotic symptoms. However, Siskind et al (2016) found clozapine to be superior to other anti-psychotics for positive symptoms. Factors influencing the difference in results included pair-wise vs network methodology and sensitivity analyses of pharmaceutical industry support. Of note, only 40% of people with TRS responded to clozapine. Clozapine’s effectiveness for reducing hospitalisations was significant, with a relative risk of 0.74 (95%CI 0.69–0.80).

**Discussion:**

There are a lack of recent non-industry funded randomised control trials of clozapine compared to SGAs, which hinders an equivocal statement about the superiority of clozapine for total psychotic symptoms. However, there is evidence to suggest that clozapine is superior to other antipsychotics, including SGAs, for positive symptoms. In terms of effectiveness, initiation of clozapine can reduce the proportion of people hospitalised and reduce bed days. Use of clozapine needs to be balanced against its adverse drug reaction profile. There remains a need for more effective treatments for TRS, and biomarkers to identify TRS.

